# TGF-β1 Pretreatment Improves the Function of Mesenchymal Stem Cells in the Wound Bed

**DOI:** 10.3389/fcell.2017.00028

**Published:** 2017-04-04

**Authors:** Deepraj Ghosh, Daniel J. McGrail, Michelle R. Dawson

**Affiliations:** ^1^Department of Molecular Pharmacology, Physiology, and Biotechnology, Brown UniversityProvidence, RI, USA; ^2^Department of Systems Biology, University of Texas MD Anderson Cancer CenterHouston, TX, USA; ^3^School of Engineering, Brown UniversityProvidence, RI, USA

**Keywords:** wound healing, mesenchymal stem cells (MSCs), transforming growth factor—β1 (TGF-β1), adhesion, migration, focal adhesion kinase (FAK)

## Abstract

The wound healing process initiates after injury to a tissue and involves a series of orchestrated events to minimize the invasion of foreign matters such as bacteria and efficiently regenerate the damaged tissue. A variety of cells must be recruited to the tissue during wound healing. However, this process is severely disrupted in patients suffering from chronic illness, including diabetes, leading to impaired healing or non-healing wounds. Current avenues of treatment include negative-pressure therapy, wound debridement, growth factor replacement, and cell-based therapies. Among these therapies, mesenchymal stem cells (MSCs) delivery to the wound holds a very high promise due to the innate abilities of MSCs that include immunogenicity, plasticity, and self-renewal. Bone marrow derived MSCs have been shown to promote more rapid wound healing by increased cytokine production in diabetic mice. However, the lack of understanding of the mechanical and chemical interaction of the transplanted MSCs with the factors present in the regenerative niches limits their efficacy in the wound bed. In this study, we sought to understand how the changes in MSC biochemical and biophysical properties can affect their function *in vitro* and *in vivo*. We demonstrate that pretreatment of MSCs with the mechano-stimulatory soluble factor transforming growth factor (TGF-β1), which is highly expressed in injury sites, improves wound closure in a syngeneic murine wound model. This improved wound closure correlated with increased invasion into the wound bed. *In vitro* studies demonstrated that TGF-β1 pretreatment expedited wound closure by increasing adhesion, traction force, and migration even after removal of the stimulus. Furthermore, this response was mediated by the cytoskeletal protein focal adhesion kinase. Taken together, this study suggests that defined chemical stimuli can benefit site specific adaptability of MSCs to improve their function and therapeutic usefulness.

## Introduction

Skin is the outermost tissue that acts as a barrier against foreign matter incursion into the body. Injury to skin initiates the wound healing process, which includes three distinct but temporally overlapping series of events namely, inflammation, proliferation, and remodeling (Diegelmann and Evans, 2004; Guo and Dipietro, 2010; Takeo et al., 2015). During inflammation, platelets secrete a host of soluble factors to attract neutrophils and macrophages, and these cells subsequently remove the contaminants from the wound bed and protect wound site from further infection. Once the inflammation process subsides, the healing process enters the proliferation stage which is characterized by fibroblast migration, matrix deposition and granulation tissue formation, angiogenesis, and re-epithelialization. Finally, the healing process moves to the remodeling phase which requires simultaneous deposition and degradation of extracellular matrix including collagen isoforms, to form scar tissue and rebuild the native architecture of the skin. However, the progress of the healing events are severely disrupted in elderly patients and patients suffering from chronic illness, including diabetes, ischemia, or hypertension, leading to non-healing chronic wounds (Gosain and DiPietro, 2004; Brem and Tomic-Canic, 2007; Guo and Dipietro, 2010). Chronic wounds are caught in a state of constant inflammation which disrupts the initiation of later phases and it is characterized by impaired production of cytokines and growth factors and reduced angiogenesis (Werner and Grose, 2003; Barrientos et al., 2008; Lazaro et al., 2016). Nearly 1% of the population in developed countries suffers from chronic wounds (Simka and Majewski, 2003; Gottrup, 2004). In United States alone, chronic wounds affect 6.5 million people and incur an estimated annual cost of USD 25 billion (Sen et al., 2009). Conventional treatment of the chronic wounds includes surgical debridement, biological dressing, pressure offloading, and topical growth factors. However, these methods provide very limited results in most cases (up to 50%) and new therapies are required to overcome the current challenges of chronic wound management (Greer et al., 2013; Dreifke et al., 2015; Rowan et al., 2015; Sessions et al., 2017). Recent studies have indicated that cell based therapies can provide a suitable alternative treatment method for multiple pathological conditions including, wound healing (Chen et al., 2012; Rodriguez-Menocal et al., 2015; Duscher et al., 2016).

Mesenchymal stem cells (MSCs) are multipotent cells that actively participate in tissue repair and regeneration *in vivo*. MSCs can be isolated and expanded with relative ease and their immunogenicity sets them as the ideal candidate for both autologous and allogeneic transplantation in wound healing (Bobis et al., 2006; Murphy et al., 2013). Both preclinical studies with animal models and clinical studies have demonstrated effectiveness of MSCs in accelerating chronic wound healing (Wu et al., 2007a; Jackson et al., 2012; Isakson et al., 2015; Kim et al., 2015; Kirby et al., 2015). MSCs added (topical delivery) or recruited (systemic delivery) to the wound bed are believed to participate in healing process by one of the following mechanisms: (1) releasing paracrine factors e.g., cytokines, and (2) differentiating into cell phenotypes e.g., keratinocytes, required for regeneration (Wu et al., 2007b; Chen et al., 2008; Sasaki et al., 2008; Kuo et al., 2016). Despite the recent progress in evaluating MSC based therapies for wound healing, the distinct interaction of MSCs with the mechanical and chemical cues present in wound bed is not fully understood and this currently limits the extensive use of MSCs in clinical capacity. Previous studies have reported that wound stiffness increases dramatically during the healing process and modifies the function of underlying cells, e.g., fibroblasts, in both normal and pathological conditions (Goffin et al., 2006; López et al., 2010; Liu et al., 2010a; Chao et al., 2013; Klingberg et al., 2013; Darby et al., 2014). In addition to the biochemical signals present in the wound milieu, such mechanical cues e.g., matrix elasticity, can also affect the MSC function profoundly (Engler et al., 2006; Kilian et al., 2010; Tse and Engler, 2011; MacQueen et al., 2013). Studies with polyacrylamide (PA) substrate of varying elasticity have reported stiffness dependent migration of cells in the context of wound healing (Ng et al., 2012; Raab and Discher, 2016). It is now widely accepted that substrate stiffness can influence a myriad of fundamental properties and functions of diverse cell types including MSCs (Discher et al., 2005, 2009; Eyckmans et al., 2011; Janmey and Miller, 2011; Charras and Sahai, 2014; Humphrey et al., 2014). In this study, we sought to understand the effect of inducing phenotypical changes (including mechanical properties) in MSCs on its function in wound bed and wound healing rate. We have previously characterized the mechanical and chemical response of MSCs *in vitro* to soluble factors present in regenerative niches (Ghosh et al., 2014). Transforming growth factor β1 (TGF-β1), a pleiotropic protein belonging to the TGF-β superfamily, regulates a myriad of cell functions including, proliferation, differentiation, adhesion, migration, and apoptosis (Massagué, 1998; Heldin et al., 2009; Watabe and Miyazono, 2009). TGF-β1 plays a significant role throughout the phases of wound healing (Gilbert et al., 2016). Our studies with soluble factor TGF-β1 provided enhanced mechanical response with cytoskeletal remodeling and stiffening of MSCs (Ghosh et al., 2014). TGF-β1 treated MSCs also provided molecular response to indicate adhesive strengthening, ECM remodeling and differentiation (Ghosh et al., 2014). This study sought to understand if TGF-β1 pretreatment induced modification in MSC phenotype can alter their *in vitro* and *in vivo* behavior. We hypothesized that migrating MSCs that disseminated throughout the wound bed would contribute to the formation of granulation tissue, which would constrict the wound for more rapid wound closure. Improved MSC migration could also improve the spatial and temporal activity of growth factors and cytokines since they were secreted from MSCs that disseminated throughout the wound tissue. Injection of TGF-β1 pretreated MSCs at the periphery of skin wounds resulted in increased wound closure rates compared to control MSCs. TGF-β1 pretreated MSCs also demonstrated greater distribution toward the center of the wound compared to control cells. The persistent characteristics of TGF-β1 pretreated cells can be beneficial for treatment of chronic wounds, where cell functions are arrested due to rapid degradation of soluble factors. To better understand the effects of TGF-β1 pretreatment, we performed multiple *in vitro* functional analyses of MSCs up to 24 h after removal of initial stimulus. TGF-β1 treatment resulted in dramatically elongated morphology and this phenotype was maintained even after 24 h of removal of the stimulus. Similarly, TGF-β1 pretreated cells sustained the enhanced surface expression of α_v_, β_1_, and β_3_ integrins as determined by flow cytometry and subsequently displayed higher adhesive strength compared to control cells. To better understand the initial cell attachment process, we used 34 kPa PA substrates that closely match the stiffness of the wound bed (Goffin et al., 2006; Discher et al., 2009). TGF-β1 pretreated cells adhered and spread more efficiently on the PA substrates and generated significantly higher traction forces. TGF-β1 pretreatment also enhanced the soluble factor-mediated migration of MSCs. Additionally, using small molecule inhibitors to disrupt certain well known pathways associated with cell functions, we found that focal adhesion kinase (FAK) signaling is key for enhanced performance of TGF-β1 pretreated cells.

## Materials and methods

### Materials

IMDM, DMEM, L-glutamine, penicillin-streptomycin, and trypsin were purchased from Mediatech and fetal bovine serum (FBS) was purchased from Atlanta Biologicals. Recombinant human TGF-β1, PDGF and IGF-I proteins and flow cytometry antibodies were purchased from Biolegend. Recombinant proteins were solubilized in phosphate buffered solution (PBS) containing 1% bovine serum albumin (BSA) and stored in −80°C as per manufacturer's recommendation. All other reagents were purchased from VWR unless otherwise specified.

### MSC isolation and culture

Murine MSCs were isolated from the bone marrow of 6–10 weeks old adult male Balb/C mice (Charles River Laboratories, Wilmington, MA) and cultured in normal growth media (IMDM media supplemented with 20% FBS, 2 mM L-glutamine, 100 U/ml penicillin, and 100 U/ml streptomycin). Purified MSCs between passages 2–6 were used for all studies after thorough characterization (Supplementary Figure 1). All animal studies were approved by the Institutional Animal Care and Use Committee at Georgia Institute of Technology (PHS Assurance Number 3822-01).

### Soluble factor pretreatment

Soluble factor dilutions were created from aliquots in serum-free DMEM or IMDM immediately before use. Based on literature review and our previous studies, TGF-β1 concentration of 5 ng/ml was used for treatment of MSCs (Ghosh et al., 2014). Initially, MSCs were pretreated with serum-free control media (CM), and 5 ng/ml TGF-β1 (diluted in serum free media) for 24 h. Afterwards, the stimulations were removed and both control and pretreated cells were moved to serum free or specific differentiation induction media to determine the effects of pretreatment on MSC functions. To avoid confusion, the removal of growth factor stimulus was assigned as t_0_; whereas the time points for each experiment were indicated as *t*_0_ + *t* h. For example, centrifugation based adhesion assays described in this section were carried out at *t*_0_ + 24 h.

### Wound preparation and mesenchymal stem cell transplantation

An *in vivo* punch biopsy wound healing model was used to determine the effects of MSC pretreatment on wound healing (McGrail et al., 2013). To account for mouse to mouse variation, all mice had one wound injected with control media pretreated MSCs and one TGF-β1 pretreated MSCs (Supplementary Figure 2). Briefly, hair was removed from the dorsal surface of anesthetized (100 mg/kg ketamine and 10 mg/kg xylazine or isoflurane gas) 12-week old male Balb/C mice by shaving and Nair hair removal. One 5 mm full-thickness skin wound was made on each side of the dorsal midline using a punch biopsy tool (to trace the wound perimeter) and iris scissors (to remove the tissue). Concurrently, pretreated MSCs were detached and labeled with the lipophilic tracer dye DiD (Invitrogen). Mouse mesenchymal stem cells (5.0 × 10^5^) suspended in a small volume of PBS solution (~100 μl) were injected (30-gauge needle) intradermally at the wound periphery of anesthetized mice. All animal studies were approved by the Institutional Animal Care and Use Committee at Georgia Institute of Technology (PHS Assurance Number 3822-01).

### Wound healing analysis

After seven days, animals were sacrificed and wound tissues were collected to image the fluorescently labeled MSCs. Wound tissues were stained with DAPI to highlight the nuclei and were then mounted on slide for imaging. The wound bed was imaged using Nikon Eclipse Ti inverted fluorescence microscope and an image of the entire wound area was created by stitching together all the individual images in Nikon Elements. The images were further analyzed by quantifying the fluorescence intensity across the distance between the centroid of the injection sites as previously discussed (McGrail et al., 2013).

### Morphological analysis

Control and pretreated MSCs were cultured for 24 h after the removal of stimulus (*t*_0_ + 24 h) and were stained with crystal violet. Cells were then imaged with stereoscope microscope and Motic camera. Cell borders were traced manually and cell shape factors, defined as 4^*^π^*^Area/ (Perimeter) ^2^, were determined using Image J.

### Centrifugal force based adhesion assay

Briefly, control and pretreated MSCs were trypsinized and labeled with Calcein AM. Then the cells were seeded in an uncoated 96-well plate in serum-free media. At 24 h after stimulus was removed (*t*_0_ + 24 h), an initial fluorescence reading was recorded. Cells were detached by centrifuging inverted plates at 500 × g for 3 min before recording a final fluorescence reading. The adherent fraction was determined by normalizing the final florescence values with the initial pre-spin values (McGrail et al., 2013).

### Flow cytometry

Briefly, both treated and untreated cells were cultured for 24 h after the removal of stimulus (*t*_0_ + 24 h), detached from surface, centrifuged, and suspended in 100 μl cold FACS buffer (2% FBS, 1mM EDTA in PBS) with one of the following anti-mouse antibodies (dilutions in parentheses): FITC-CD29 (1:100), PE-CD61 (1:100), PE-CD51 (1:20), AF-647-MVCAM1/CD106 (1:200). Following the incubation, MSCs were washed and fixed in 2% paraformaldehyde. Samples were run on a BD LSR-II flow cytometer to capture at least *n* = 50,000 events per sample.

### Fabrication of polyacrylamide (PA) substrates

PA hydrogels were used as an elastic 2D cell culture platforms due to extensive characterization of their mechanical properties that can be easily tuned by controlling crosslinker concentration(Tse and Engler, 2010; Caliari and Burdick, 2016; Polacheck and Chen, 2016). PA substrates were synthesized based on the protocol described before (Tse and Engler, 2010). Briefly, glass coverslips were activated using 3-aminopropyltrimethoxysilane and a mixture of the acrylamide and bis-acrylamide solution (10% acrylamide to 0.3% bis-acrylamide) was polymerized on the activated glass coverslips (Young' modulus (E) ~34 kPa). Substrates were coated with type I collagen solution (0.2 mg/ml) before cell culture.

### Adhesion assay

Control and pretreated MSCs were trypsinized and labeled with a transmembrane fluorescent viability marker, Calcein AM (Anaspec), in HBSS containing divalent ions Ca++ and Mg++. Then the cells could adhere for 3 h after stimulus was removed (*t*_0_ + 3 h) in HBSS before taking an initial florescence reading in a DTX-800 Multimode Detector plate reader. A final reading was taken after removing non-adherent cells by washing with HBSS to determine the adherent fraction.

### Traction force microscopy

Control and pretreated MSCs were seeded on collagen-coated PA substrates embedded with 200-nm fluorescent microbeads, for 24 h. Particle displacements were determined by comparison of the embedded bead positions before and after the cells were detached from the gels and used to determine traction forces with a Fourier-transform traction force cytometry in a custom-written MATLAB routine as previously described (Sabass et al., 2008).

### Inhibitor studies

PF573228, Ly294002, and SIS3 were used to inhibit FAK, PI3K, and SMAD3 mediated signaling, respectively. Range of concentrations for all the inhibitors were determined from literature reviews and MSCs were treated with 10 μM concentration (Jeon et al., 2008; Qureshi et al., 2008; Gharibi et al., 2012; Zhang et al., 2015, 2016). For adhesion studies cells control and pretreated cells were detached and suspended in inhibitor supplemented serum free media for 2 h before seeding on a 96-well plate. Then the cells could adhere for 3 h before taking an initial florescence reading. A final reading was taken after removing non-adherent cells by washing with HBSS to determine the adherent fraction. For both motility and scratch assay cells were treated with inhibitors for 2 h before adding the migration stimulating factor PDGF (15 ng/ml).

### Motility assay

Cells were treated with control and TGF-β1 supplemented media for 24 h in 10 cm dishes. Both control and pretreated MSCs were trypsinized and seeded on collagen coated 48-well plate at a sub-confluent density for 12 h. Wells were then washed to remove detached cells before staining with Hoechst 33,342 for 30 min. Cells were then treated with soluble factors before placing them on Nikon Eclipse Ti inverted epifluorescent microscope, which was maintained at 37°C and 5% carbon dioxide throughout the experiment using an *In vivo* Scientific environmental cell chamber. Images of the nucleus were taken using DAPI filter (excitation 358 nm and emission 461 nm) at multiple points at 12 min- interval for 6–8 h at 10x magnification. The locations of cell nuclei, segmented from fluorescent images, were tracked in MATLAB to define cell traces. The cell migration coefficients and directional velocities were determined by fitting the traces to the persistent random walk model (Dickinson and Tranquillo, 1993). Briefly, mean square displacements were calculated (MSD = d^2^(τ)) from the two-dimensional tracking data and was used for fitting the following equation: (where, d = displacement, t = time, P = persistence time, and μ = migration coefficient).

$$\begin{array}{l} \langle d^{2}\left(\tau \right)\rangle =4\mu \left\{t-P\left[1-e^{-t/P}\right]\right\} \end{array}$$

### Scratch assay

Cells were cultured as a confluent monolayer on collagen coated 48-well plate and a gap was created by removing cells with a pipette tip. Wells were then washed to remove detached cells before staining with Calcein AM. Cells were then treated with soluble factors before placing them on Nikon Eclipse Ti inverted epifluorescent microscope, which was maintained at 37°C and 5% carbon dioxide throughout the experiment using an *In vivo* Scientific environmental cell chamber. FITC green channel (495 nm excitation and 515 nm emission) images were taken at multiple points at 15-min interval for 12 h at 10x magnification.

### Statistics

Each experiment was performed with 3 or more replicates, and all values expressed as the mean ± s.e.m. For comparison between two groups student *t*-test was used. One way Anova test with repeated measures was used to determine statistical significance of experiments involving more than two groups. For comparison between groups, Tukey's HSD post-test was used. Significance was reported as ^*^ (for *p* < 0.05), ^**^ (for *p* < 0.005), and ^***^ (for *p* < 0.0005).

## Results

### TGF-β1 pretreatment of MSCs enhances their distribution in wound bed

**Figure 1:**
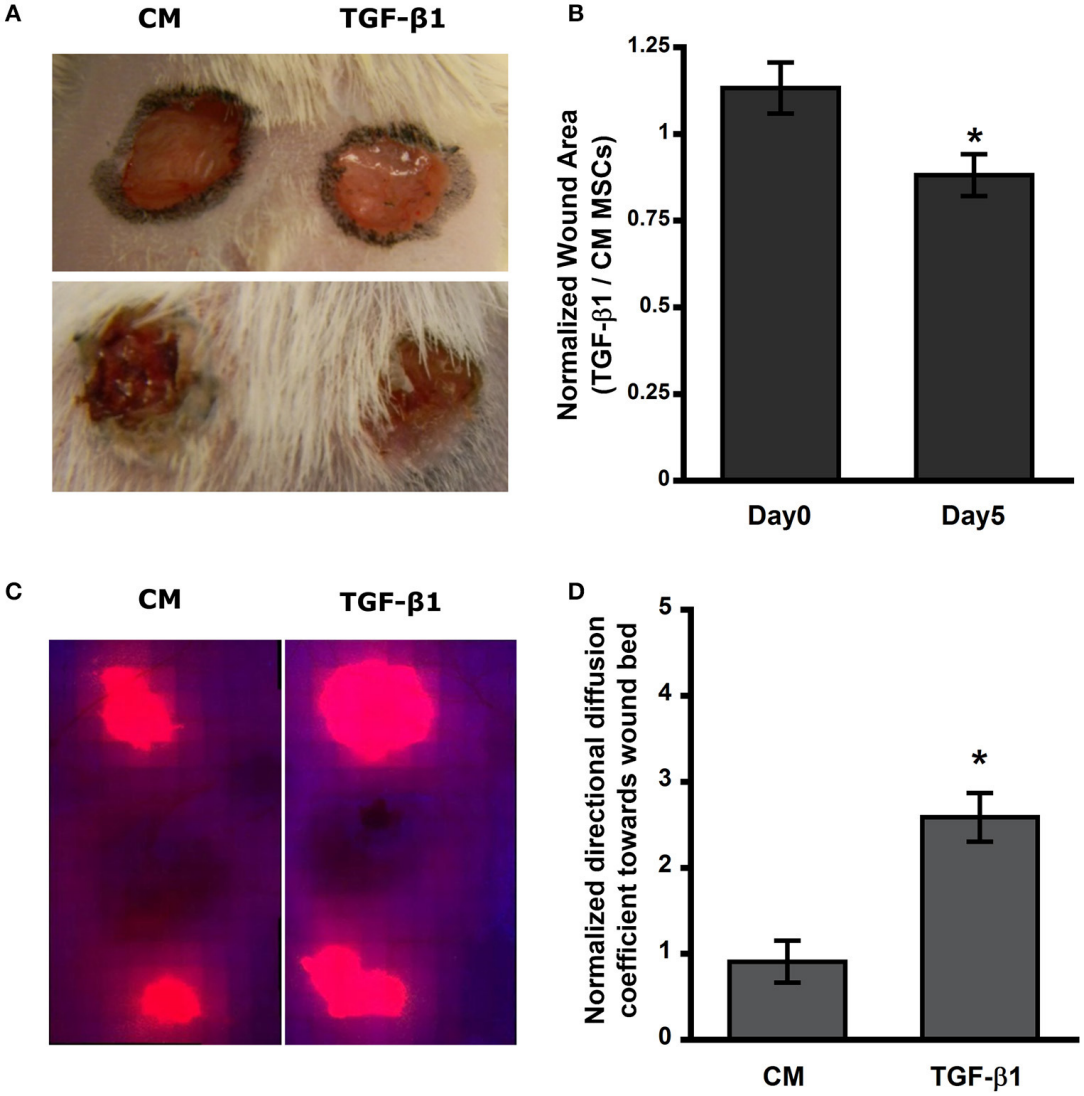
**Transforming growth factor–β1 (TGF-β1) pretreated mesenchymal stem cells (MSCs) migrate and distribute efficiently in the wound bed and improve wound closure rate in Balb/C mice. (A)** Full thickness skin wound on Balb/C mice at day 0 and 5. **(B)** Open area of TGF-β1 pretreated MSCs injected wound normalized to its respective control. **(C)** Thresholded fluorescence images of skin tissue showed enhanced distribution of MSCs toward center of the wound. **(D)** Quantification of directed distribution of MSCs toward the center of the wound (*n* = 3). Values given as mean ± s.e.m.; significance is indicated as ^*^(*p* < 0.05).

MSCs have previously been used to increase the rate of wound closure in full thickness skin wounds; however, untreated MSCs used in these studies resulted in a modest change in the rate of wound healing and long-term engraftment of MSCs was not clearly established. We used a syngeneic mouse wound healing model to evaluate the effect of TGF-β1 pretreatment on dissemination of MSCs *in vivo*. Fluorescently labeled MSCs (control and TGF-β1 pretreated) were injected at the periphery of full thickness wounds in Balb/C mice. At day 0 the ratio of TGF-β1 to control MSCs treated wounds for all the mice were close to 1 suggesting similar areas; however, after day 5 normalized wound areas were ~15% reduced suggesting significant wound closure rate with TGF-β1 pretreated MSCs compared to untreated cells (Figures 1A,B, Supplementary Figure 3). By day 7, wounds were mostly closed (Supplementary Figure 4). However, by analyzing the labeled MSCs in the wound bed we found that TGF-β1 pretreated cells displayed more efficient distribution toward the center of the wound (Figures 1C,D).

### Sustained morphological changes at 24 h after TGF-β1 pretreatment

**Figure 2:**
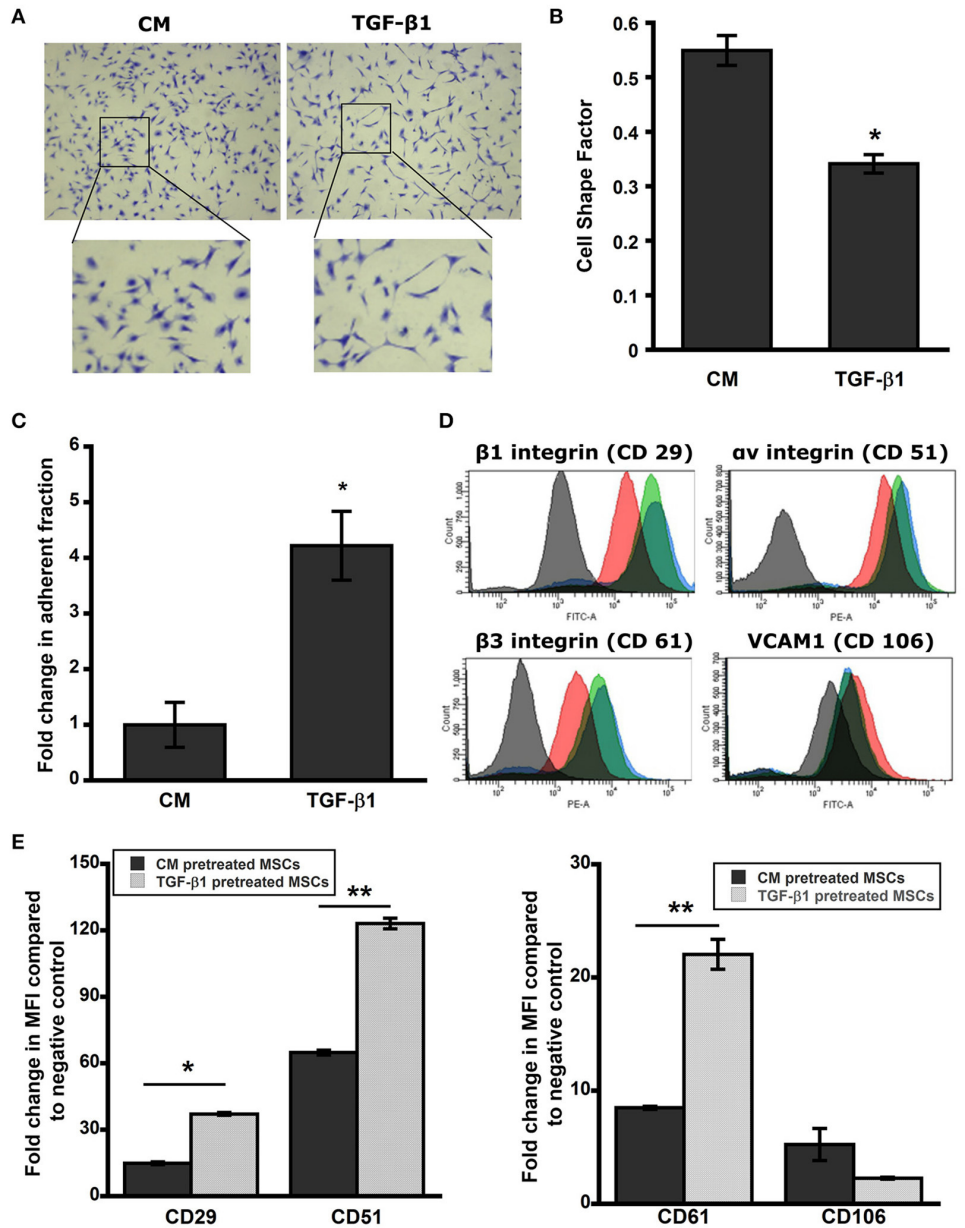
**Pretreated MSC maintain elongated phenotype. (A)** Brightfield images of pretreated MSCs after 24 h stained with crystal violet. TGF-β1 pretreated MSCs sustained elongated morphology in serum free media for a period of 24 h. **(B)** Cell shape factor (CSF) was determined by analysis of bright field images with Image J. CSF was used to characterize the elongation of the cell, with a shape factor of 1 indicating a perfect circle and 0 indicating a straight line. Results are reported as average ± s.e.m. (*n* = 2). **(C)** The centrifuge-based adhesion assay was used to determine the effect of soluble factor pretreatment on the adhesion of MSCs on uncoated surface. **(D)** TGF-β1 pretreated MSCs maintain surface adhesion characteristics. Histograms from flow cytometry were analyzed using FACS-DIVA for mean fluorescence intensity (MFI). Expressions of surface integrins αv (CD51), β1 (CD 29), and β3 (CD61) were increased significantly in TGF-β1 pretreated cells compared to the control; whereas TGF-β1 pretreatment reduced VCAM1 (CD106) expression significantly compared to the control. **(E)** Analysis of cell surface adhesion molecules α_v_ (CD51-PE), β_1_ (CD29-FITC), and β_3_ (CD61-PE) integrins, and VCAM-1 (CD106-FITC) using flow cytometry after 24 h of removal of stimulus. Black and red indicates negative and control cell population; whereas green and blue represent TGF-β1 pretreated cells at immediately (*t*_0_) and 24 h (*t*_0_ + 24 h) after the removal of stimulus respectively. TGF-β1 pretreated cells exhibit higher integrin expression and lower VCAM-1 expression at 24 h after removal of the stimulus compared to control cells. Results are reported as average. (*n* = 2). Values given as mean ± s.e.m.; significance is indicated as ^*^(*p* < 0.05), ^**^(*p* < 0.005).

We have previously demonstrated that MSCs undergo dramatic elongation in response to TGF-β1 treatment (Ghosh et al., 2014). To examine the persistent effect of TGF-β1 on cell shape, pretreated MSCs were detached and reseeded on a new surface (TCP) with serum free media in the absence of stimulus for 24 h (*t*_0_ + 24 h). Cells were fixed and stained with crystal violet to analyze cell morphology using a cell shape factor (CSF) which varies from 0 for a line to 1 for a perfect circle. TGF-β1 pretreated cells retained the elongation effect even after 24 h (*t*_0_ + 24 h; Figure 2A). Control MSCs retained their spindle shape leading to CSF close to 0.5. Still the cell shape factor of TGF-β1 pretreated cells was significantly lower (~0.3) than its control counterpart indicating dramatic elongation (*p* < 0.05; Figure 2B).

### TGF-β1 pretreatment enhances integrin expression and adhesive strength of MSCs

Next we sought to analyze the sustained effect of pretreatment on adhesive strength of MSCs using a centrifugation force based assay at 24 h (*t*_0_ + 24 h). TGF-β1 pretreated cells maintained their higher adhesivity to ECM and exhibited a 4.2-fold increase in the adherent fraction compared to control (Figure 2C). To better elucidate the role of cell surface adhesion molecules that control cell-cell and cell-ECM interaction, we analyzed integrin subunits α_*v*_ (CD51), β_1_ (CD29), β_3_ (CD61), and vascular cell adhesion molecule-1 (VCAM-1, CD106) using flow cytometry at this time point (*t*_0_ + 24 h; Figure 2D, Supplementary Figure 5). TGF-β1 pretreated cells displayed significantly upregulated expressions of the integrins while reducing VCAM-1 expression (Figure 2D). Mean fluorescence intensity (MFI) analysis of histograms further confirmed the trends of integrin upregulation and VCAM-1 downregulation due to TGF-β1 pretreatment (Figure 2E). This result correlated with the observed adhesive strengthening response.

### Pretreated MSCs maintain adhesive phenotype on wound-mimetic substrates

**Figure 3:**
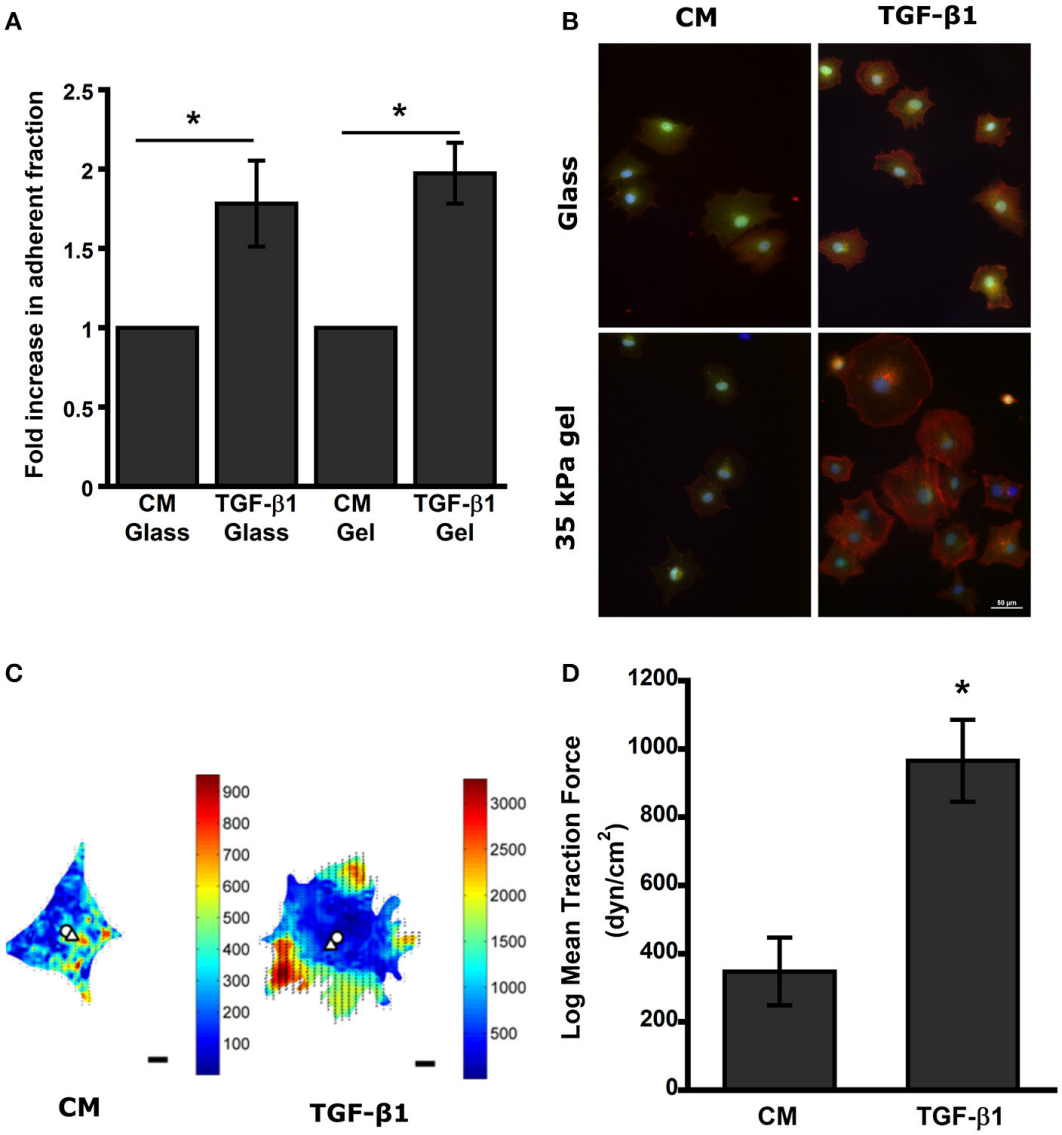
**TGF-β1 pretreated MSCs display higher adhesivity and traction force after removal of stimulus. (A)** TGF-β1 pretreated cells display higher adhesivity on both glass and gel. **(B)** Pretreated MSCs (CM vs. TGF-β1) plated on glass and PA gels were washed after 3 h to remove detached cells. (Red-Actin, Blue- DAPI, Green- Calcein AM). **(C)** Traction force heat map of CM and TGF-β1 treated MSCs, **(D)** TGF-β1 pretreated MSCs exerted higher traction force compared to control after 24 h' removal of stimulus. Values given as mean ± s.e.m.; significance is indicated as ^*^(*p* < 0.05).

To better understand the difference between pretreated MSCs and control during the initial stages of attachment in the wound bed, we used compliant semi-flexible PA substrate with stiffness (E ~34 kPa) like fibrotic skin wound (Goffin et al., 2006; Discher et al., 2009). TGF-β1 pretreated MSCs seeded on both collagen coated glass and PA gel substrates for 3 h (*t*_0_ + 3 h) exhibited >1.7-fold increase in adherent fraction compared to control (*p* < 0.05; Figure 3A). Furthermore, F-actin staining with Phalloidin revealed that the TGF-β1 pretreated cells spread more rapidly and display a well-defined cytoskeletal organization compared to its untreated counterparts (Figure 3B). We hypothesized that previously seen change in integrin expression may alter the traction force exerted by the MSCs on PA substrates. At 24 h (*t*_0_ + 24 h) traction force maps of TGF-β1 pretreated MSCs displayed very different force distribution compared to control cells (Figure 3C). Pretreated MSCs displayed very high forces localized around the edges without any apparent polarization. Also, the magnitude of overall traction forces exerted by the TGF-β1 pretreated cells was significantly higher compared to the control cells (Figure 3D).

### TGF-β1 pretreated MSCs displayed enhanced cell motility *in vitro*

**Figure 4:**
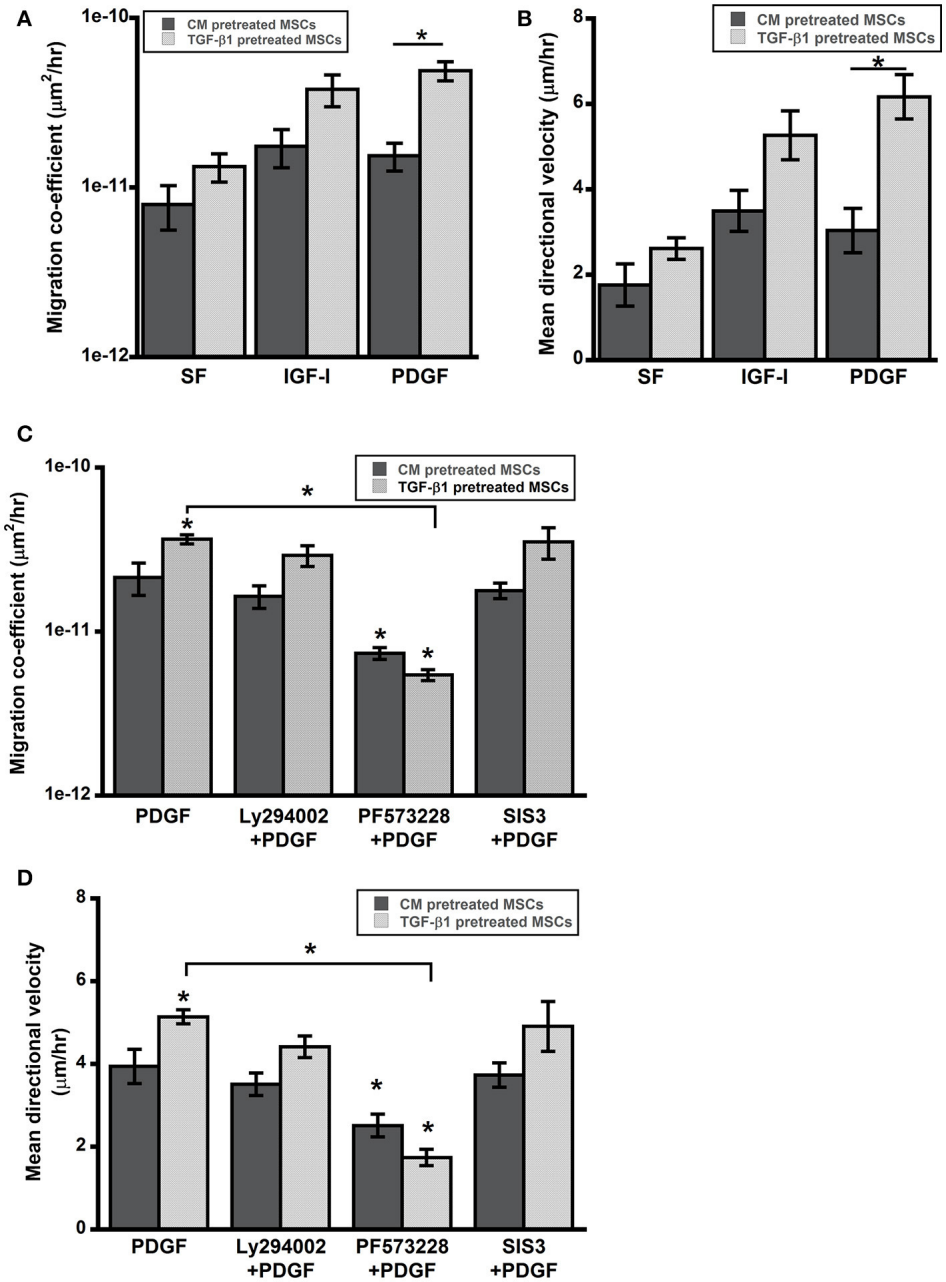
**TGF-β1 pretreated MSCs displayed enhanced cell motility. (A,B)** Cell motility of pretreated MSCs was evaluated in presence of pro-migratory factors PDGF-BB and IGF-I (present in wound bed). **(C,D)** Enhanced motility of TGF-β1 pretreated MSCs in presence of PDGF (15 ng/ml) was abrogated by FAK inhibition (PF573228) but not by PI3K (Ly294002) or SMAD3 (SIS3) inhibition. Values given as mean ± s.e.m.; significance is indicated relative to control unless otherwise noted, ^*^(*p* < 0.05).

Control and pretreated MSCs were seeded on 48-well plate to evaluate the effect of soluble factors on cell motility. In presence of pro-migratory soluble factors PDGF (15 ng/ml) and IGF-1 (30 ng/ml), the migration coefficients were determined by tracking the cell nuclei. Both PDGF and IGF-1 enhanced random cell migration speed of MSCs; however, significant increase in migration was observed for TGF-β1 pretreated cells compared to control (Figures 4A,B).

### FAK signaling is essential for adhesive strengthening and enhanced motility

**Figure 5:**
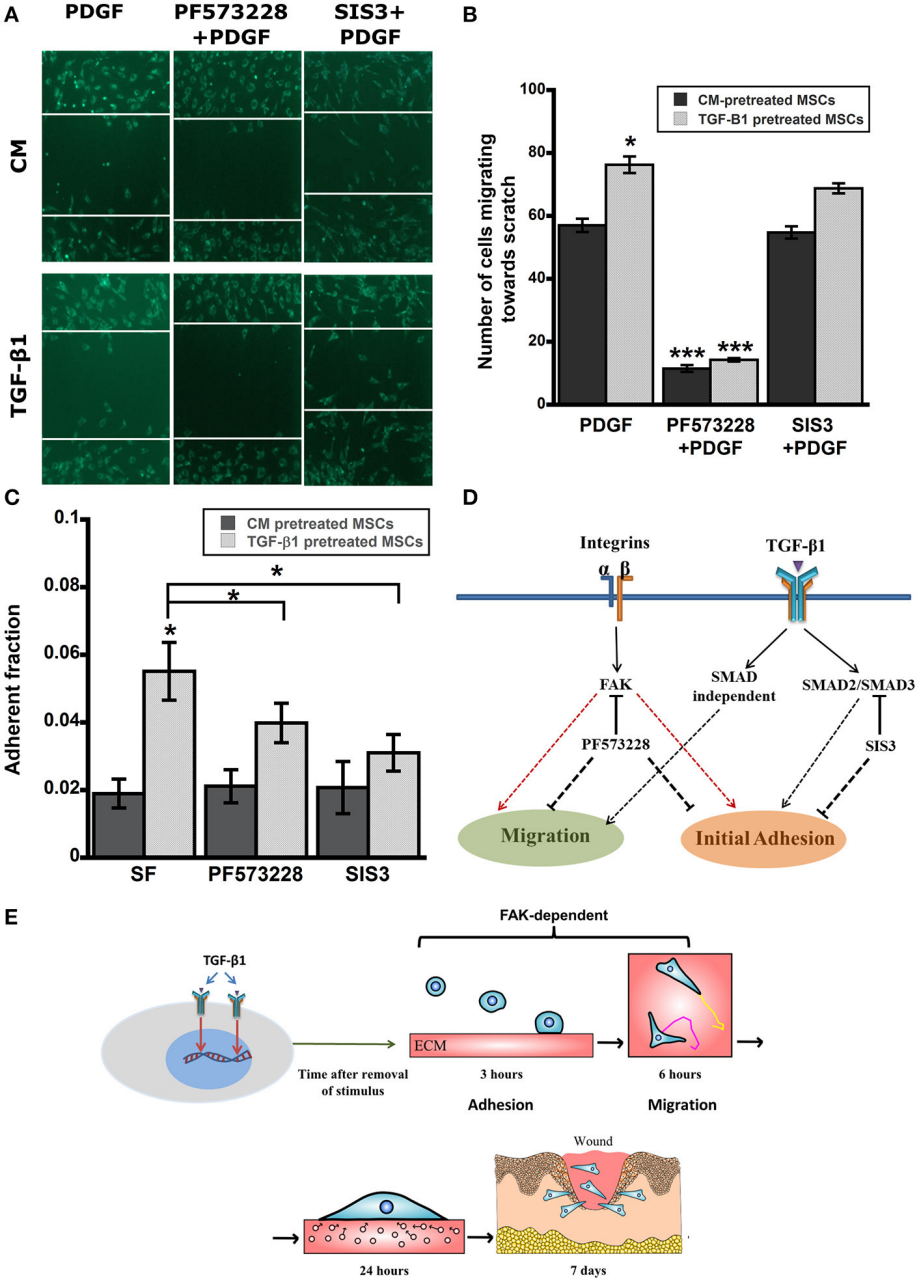
**(A)** Scratch assay was used to evaluate directed migration of pretreated MSCs in presence of inhibitors (12 h). **(B)** TGF-β1 pretreated MSCs displayed higher directed cells migration toward scratch in a SMAD2/3 independent but FAK dependent manner. **(C)** Enhanced Adhesive strength of TGF-β1 pretreated MSCs was both FAK and SMAD3 dependent. Pretreated MSCs (CM vs. TGF-β1) plated on TCP were washed after 3 h to remove detached cells. **(D)** A simplified diagram demonstrating the effects TGF-β1 pretreatment on adhesion and migration via integrin-FAK and SMAD pathways. **(E)** Proposed mechanism of TGF-β1 pretreated MSCs leading toward improved *in vitro* functions and enhanced *in vivo* wound healing. Values given as mean ± s.e.m.; significance is indicated relative to control unless otherwise noted, ^*^(*p* < 0.05), ^***^(*p* < 0.0005).

TGF-β1 pretreatment had profound effects on *in vitro* function of MSCs including, adhesion and migration. TGF-β1 regulates cell functions via both canonical and non-canonical pathways. To further elucidate roles of key signaling pathways in enhanced cell functions, we blocked following pathways using small molecule inhibitors: (1) integrin-FAK signaling using PF573228, (2) TGF-β signaling via SMAD2/3 using SIS3 and (3) growth factor mediated PI3K signaling with Ly294002. Interestingly, only FAK inhibitor PF573228 could inhibit TGF-β1 mediated enhanced migration of MSCs as the difference in migratory activity between TGF-β1 treated cells and control were completely abrogated by FAK inhibitor (Figures 4C,D). To further evaluate the effects of the inhibitors on directed migration of MSCs, we used an *in vitro* scratch assay that mimics the wound site. Cells migrated toward an artificially created gap to establish new cell-cell contact under the influence of PDGF (30 ng/ml). Consistent with the previous results FAK inhibitor treatment blocked MSC migration toward the gap (Figures 5A,B). SMAD3 inhibitor, SIS3 was not able to block TGF-β1 pretreatment dependent directed migration, implicating a SMAD2/3 independent non-canonical pathway. Next we analyzed effect of FAK and SMAD3 inhibitors on initial adhesion on TCP after 3 h and found that both pathways have significant effect in reducing adhesion (Figure 5C).

## Discussion

Wound healing is a very complex process and understanding the coordinated action of the different types of cells and niche specific soluble and insoluble factors is of utmost importance for the development of modern wound care. Growth factors and bioactive materials have been used increasingly in recent times; however, on their own, there efficacy remains limited (Greer et al., 2013). Recent studies have highlighted the benefits of using mesenchymal stem cells for wound healing that holds high hope for the future of the wound care (Wu et al., 2007a; Jackson et al., 2012; Isakson et al., 2015; Duscher et al., 2016). However, characterization of MSCs with niche specific factors is still in its early stages. In this study, we have investigated interaction between mesenchymal stem cells and TGF-β1 a growth factor that plays an important role both in normal wound healing and in fibrosis(Gilbert et al., 2016; Meng et al., 2016). The current study was designed to determine if TGF-β1 induces a persistent alteration in MSC phenotype, leading to more efficient function in the wound bed *in vivo*.

Non-healing wounds are often characterized with loss or lack of recruitment of motile cells in the wound bed that are required to close the formed gaps. Even MSCs added locally to the site often form island like structure with visible necrosis (Yang et al., 2005). Previous studies with hypoxic preconditioning and soluble factor (e.g., EGF) pretreatment have shown to enhance paracrine effects of MSCs as well as increase in cell migration *in vitro* and hypothesized that these outcomes will translate to improved wound healing *in vivo* (Lee et al., 2009; Tamama et al., 2010; Kim and Sung, 2012). We found that TGF-β1 pretreatment contributed to more efficient propagation of MSCs and increased wound closure rate *in vivo* (Figure 1). Further, analysis of the diffusion pattern of fluorescently labeled MSCs in our study revealed a 2.5-fold increase in distribution of TGF-β1 pretreated MSCs toward the center of the wound. This result agrees well with the outcomes of *in vitro* migration studies where TGF-β1 pretreated cells displayed increased directional migration compared to control cells (Figures 4, 5A,B).

To further understand the function of implanted cells, we designed a series of *in vitro* experiments to analyze the cell–matrix interactions required for adhesion and migration. Adhesion studies at 3 h (*t*_0_ + 3 h) with compliable PA gels (E ~34 kPa) representing the skin tissue stiffness indicated that TGF-β1 pretreatment increased the initial attachment to substrate (Goffin et al., 2006; Discher et al., 2009). Subsequently, a centrifuge-based assay at longer time scales (*t*_0_ + 24 h) also displayed similar trend with the measured adherent fraction. Interestingly, pretreated MSCs displayed more than 4-fold increase in the adherent cell fraction at 24 h (*t*_0_ + 3 h) compared to the 2-fold increase measured in our washing study after 3 h (*t*_0_ + 3 h; Figures 2, 3). These results would account for the adhesive strengthening due to changes in integrin expression and the secretion of native ECM proteins by MSCs on uncoated surface (at *t*_0_ + 24 h; Gallant and García, 2007; Michael et al., 2009). Previous studies have demonstrated enhanced ECM and other growth factor secretion from cells in response to TGF-β1 and transcriptional activation of the ECM genes such as collagen 1, fibronectin, and laminins were upregulated in previous microarray results (Supplementary Table 1; Ghosh et al., 2014). TGF-β1 pretreatment induced change in adhesion kinetics at 3 h may translate to more successful engraftment efficiency *in vivo*, whereas the adhesive strengthening at 24 h could increase long term survival and integration in wound bed. Additionally, TGF-β1 pretreated cells were more contractile on substrates and exerted almost 3-fold higher traction force (Figures 3C,D).

The phenotypical changes in MSCs due to TGF-β1 pretreatment that has been reported here are quite analogous to the changes associated with myofibroblast differentiation from fibroblast in wound bed with characteristics including, increase in both αv and β1 integrin expressions, increase in ECM production, and more motile phenotype (Thannickal et al., 2003; Lygoe et al., 2004; Häkkinen et al., 2011). Our analysis confirmed that TGF-β1 pretreated MSCs maintained an increased surface α_v_ and β1 integrin expression (Figures 2D,E) which has been correlated with enhanced migration both *in vivo* and *in vitro* and can be critical for the observed changes *in vivo* (Liu et al., 2010b; Veevers-Lowe et al., 2011; Saller et al., 2012; Koivisto et al., 2014). Myofibroblasts are essential for closure of the wound gap; however, chronic inflammation may hinder fibroblast recruitment to wound site and its differentiation into myofibroblast. TGF-β1 pretreated MSCs that are added locally can act as an alternative but further studies are required to characterize the functional difference between these two cell phenotypes.

All the previous results suggest that TGF-β1 pretreatment activates an integrin-FAK mediated mechanosensitive pathway and the sustained response of the cells can be related to formation of clusters between growth factor-receptors and integrins that increases and prolongs the signaling in synergy (Alam et al., 2007; Kim et al., 2011). To examine this hypothesis, three types of small molecule inhibitors were used: (1) Ly294002, a PI3K inhibitor was used as a control to ensure that the enhanced response was not due to cell proliferation, (2) PF573228 was used to block integrin mediated FAK activation, and (3) SIS3 was utilized to inhibit TGF-β1 mediated SMAD3 signaling. Ly294002 didn't alter the motility of MSCs indicating that the difference in response between treated and untreated cells is not an artifact of growth factor mediated proliferation. FAK activation has been shown to be essential for myofibroblast differentiation and wound healing and we here report that inhibiting FAK results in complete abrogation of adhesion and migration of MSCs in agreement with previous studies (Thannickal et al., 2003; Song et al., 2010). Finally, the effect of SIS3 on adhesion and migration of MSCs were quite interesting. SIS3 could block the enhanced adhesion of pretreated MSCs; however, it could not stop the directed migration of cells toward a scratch. Previous studies have correlated SMAD3 signaling inhibition with EMT switch, enhanced reepithelization and wound contraction (Ashcroft et al., 1999). In summary, the activation of FAK via integrins is essential for both SMAD3 dependent and independent signal transduction that are responsible for sustained phenotypical changes associated with TGF-β1 pretreatment *in vitro* (Figure 5D). Taken together this study suggests that TGF-β1 pretreatment of MSCs can improve their attachment rate and motility *in vivo* via activation of FAK and improves their therapeutic efficacy in wound bed (Figure 5E).

## Conclusions

The function of *ex-vivo* expanded MSC based therapeutics has been shown to be limited after reintroduction *in vivo*. This study illustrates that pretreatment of MSCs with TGF-β1 resulted in sustained improvement in migration, and adhesion even after removal of the stimulus *in vitro*. Subsequently, these pretreated cells enhanced the *in vivo* wound healing process, which may lead to improved therapeutic efficacy. Future studies with site specific factors will be used to guide new strategies for microenvironment specific MSC based therapy.

## Ethics statement

This study was carried out in accordance with the recommendations of the Institutional Animal Care and Use Committee at Georgia Institute of Technology. The protocol was approved by the Institutional Animal Care and Use Committee at Georgia Institute of Technology (PHS Assurance Number 3822-01).

## Author contributions

DG, DM, and MD designed the experiments. DG and DM performed the experiments and data analysis. DG, DM, and MD prepared the manuscript.

## Funding

Funding for this work was provided by the National Science Foundation (1032527, 1066585) and Georgia Tech and Emory Center for Regenerative Medicine (2731318). Additional funding was provided by NSF Grant DGE-0965945 (DM).

### Conflict of interest statement

The authors declare that the research was conducted in the absence of any commercial or financial relationships that could be construed as a potential conflict of interest.
