# Supplementary material for: TGF-β1 Pretreatment Improves the Function of Mesenchymal Stem Cells in the Wound Bed
Source: Front Cell Dev Biol. 2017 Apr 4;5:28. doi: 10.3389/fcell.2017.00028 (PMC5378794; doi:10.3389/fcell.2017.00028)
Supplement: Supplementary file 1 [file DataSheet1.docx]

Supplementary Material

TGF-β1 pretreatment improves the function of mesenchymal stem cells in the wound bed

Deepraj Ghosh, Daniel J. Mcgrail, Michelle R. Dawson^*^

*** Correspondence:** Dr. Michelle R. Dawson: michelle_dawson@brown.edu

# Supplementary methods

## MSC characterization

Balb/C MSCs were isolated from the bone marrow and were characterized previously^1^. Here, we investigated the effects of pretreatment on MSC phenotype. For flow cytometry, pretreated cells stained with PerCP- CD45, PE-Sca1, and APC-CD11B antibodies. For differentiation assays, cells were cultured and pretreated on 24-well plates (CM vs TGF-β1) before switching to differentiation induction media. The induction media formulation and the staining method for each differentiation studies are same as mentioned previously^1^. After 4 weeks, the cells were stained with oil red o and von kossa for adipogenesis and osteogenesis respectively.

## Microarray data analysis

Gene expression results from previously reported microarray analysis was used for gene analysis ^2^. Briefly, Affymetrix GeneChip Mouse Genome 430 2.0 microarray chips were used to perform gene expression analysis of untreated (control) and treated (TGF-β1) MSCs. The gene expression values were normalized and log_2_ transformed for further analysis. Lists of genes related to specific pathways were obtained from NCBI Biosystems and KEGG pathway databases. The fold change in gene expression is reported as log2 ratio here.

# Supplementary Figures and Tables

## Supplementary Figures


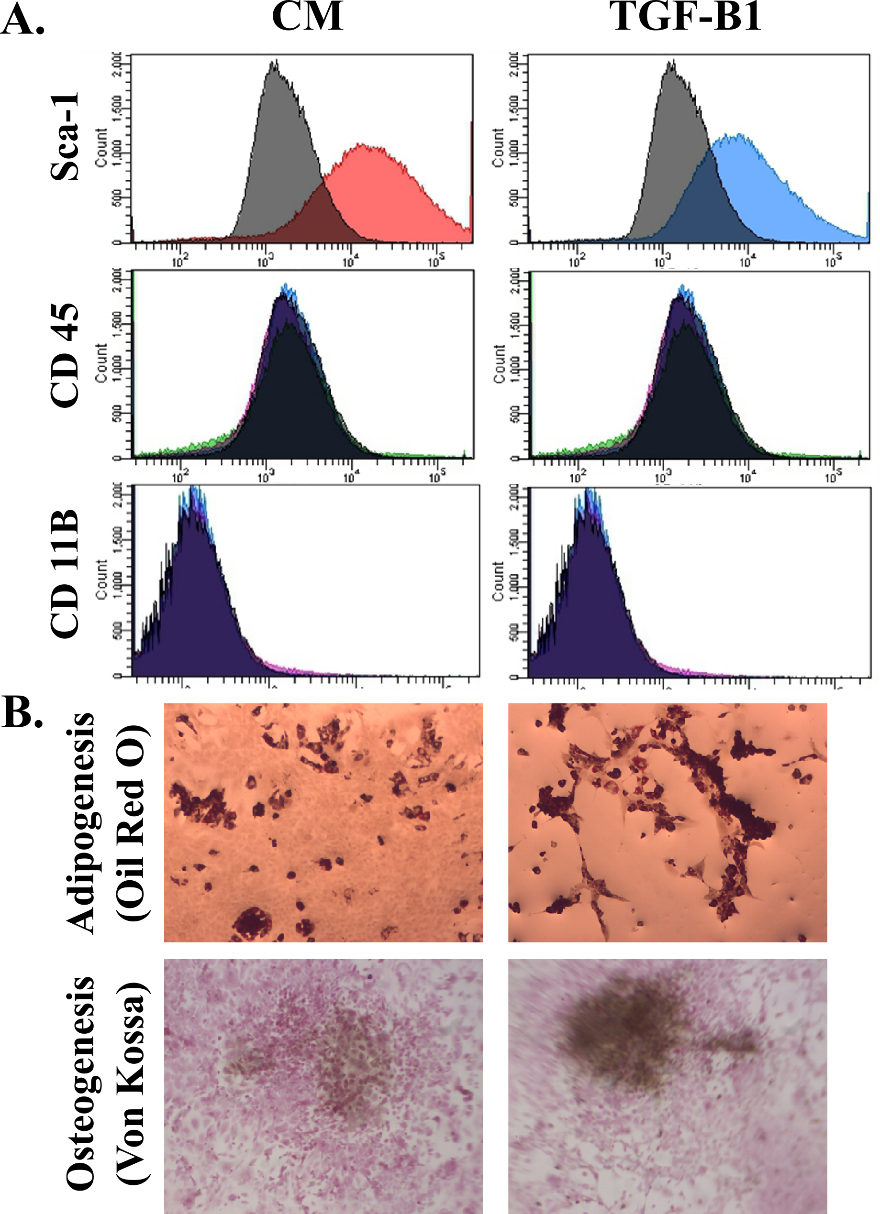


Supplementary Figure 1. MSC characterization. A. Pretreated MSCs were stained and analyzed using flow cytometry for negative (CD45, CD11B) and positive (Sca1) cell surface markers. Both MSCs population were characterized by Sca1^bright^, CD45^-^ and CD11B^-^ phenotype. B. Pretreated MSCs were treated with adipogenic and osteogenic differentiation media for 4 weeks as described previously^1^. Adipocytes and osteoblasts were positively stained with oil red o and von Kossa respectively.


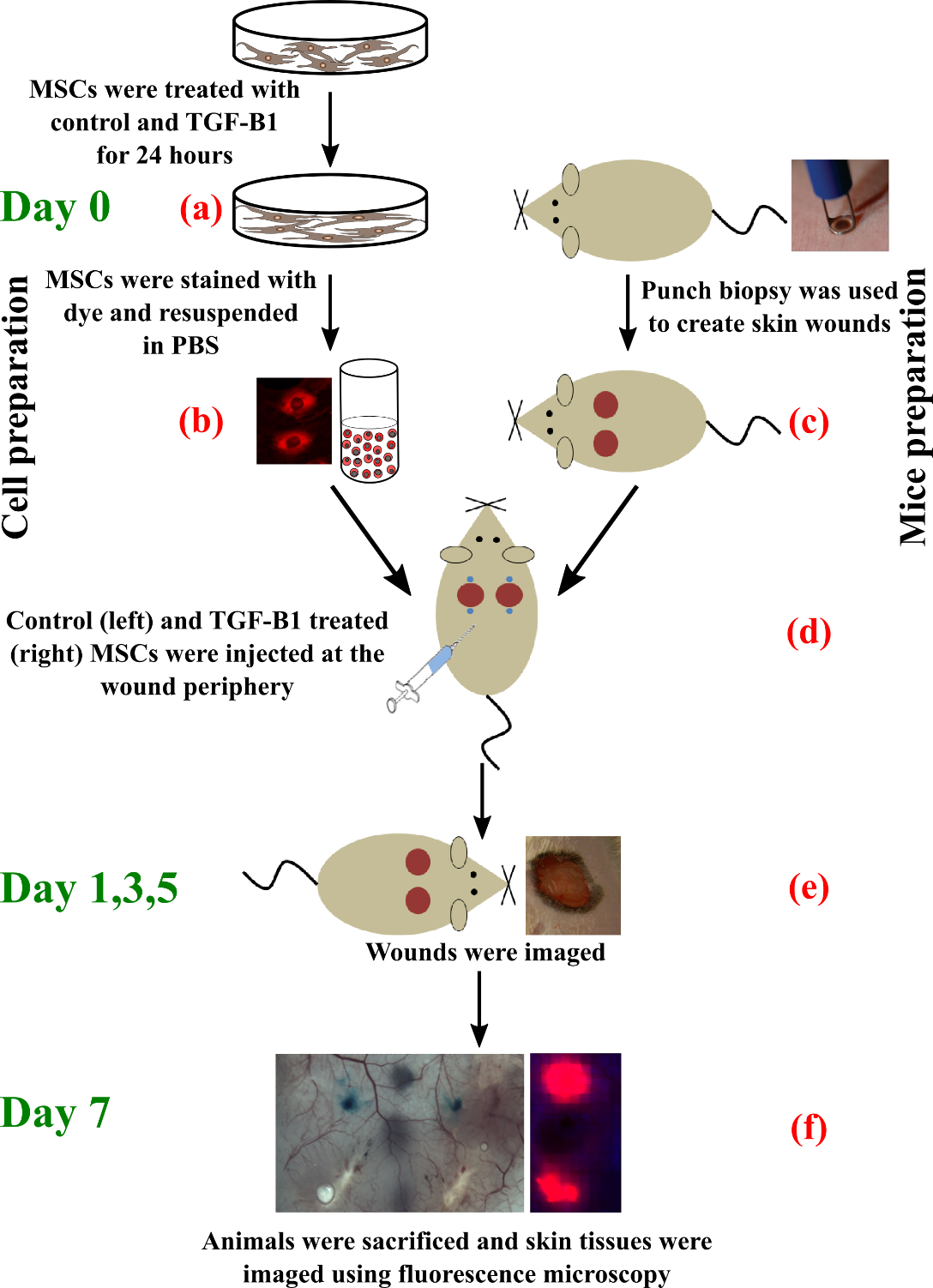


Supplementary Figure 2. Experimental design and timeline of wound healing study. Cell preparation: (a) MSCs cultured on TCP were treated with control and TGF-β1 respectively for 24 hours; (b) MSCs were stained with lipophilic tracer dye DiD and suspended in PBS (~100 µl). Mice preparation: (c) BalbC mice were anaesthetized and two dorsal full thickness wounds were created using punch biopsy (5mm.). Cell implantation *in vivo*: (d) MSCs suspended in PBS were injected intradermally at the periphery of the wound (blue dots). Wound closure rate: (e) Open wound area was imaged at days 0, 1, 3, 5 to monitor healing rate. Analysis of MSC distribution in wound bed: (f) Mice were sacrificed on Day 7 and skin tissues around the wound were collected and mounted on slide to image on a microscope. A composite image of the total area for each wound encompassing both injection points (bright red) was acquired to analyze MSC distribution.


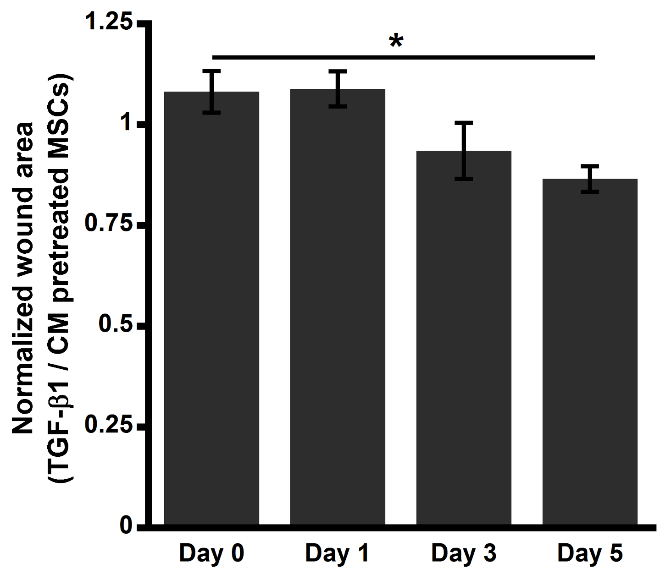


Supplementary Figure 3: Relative wound closure rate. Open wound area ratio of TGF-β1 pretreated MSCs injected wound to control MSCs treated wound decreases significantly on Day 5.


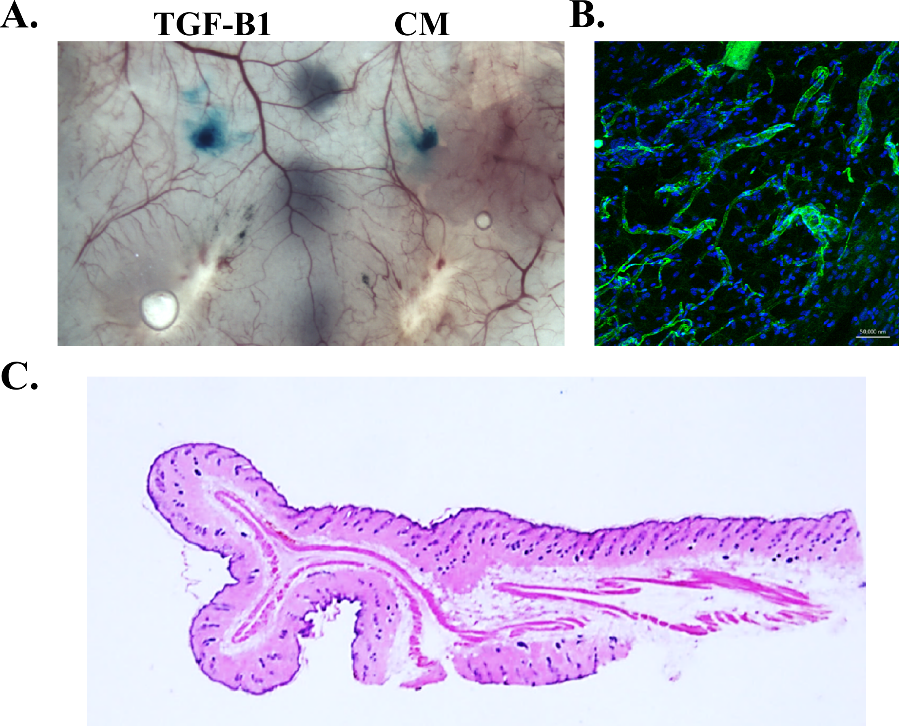


Supplementary Figure 4. Healing and revascularization of wounds. (A) Stereoscopic image showing re-vascularization of full-thickness wounds. (B) Immunostaining for CD31 to verify presence of blood vessels (Green- CD31-FITC, Blue-DAPI). (C) H&E staining showing healing of wound bed.


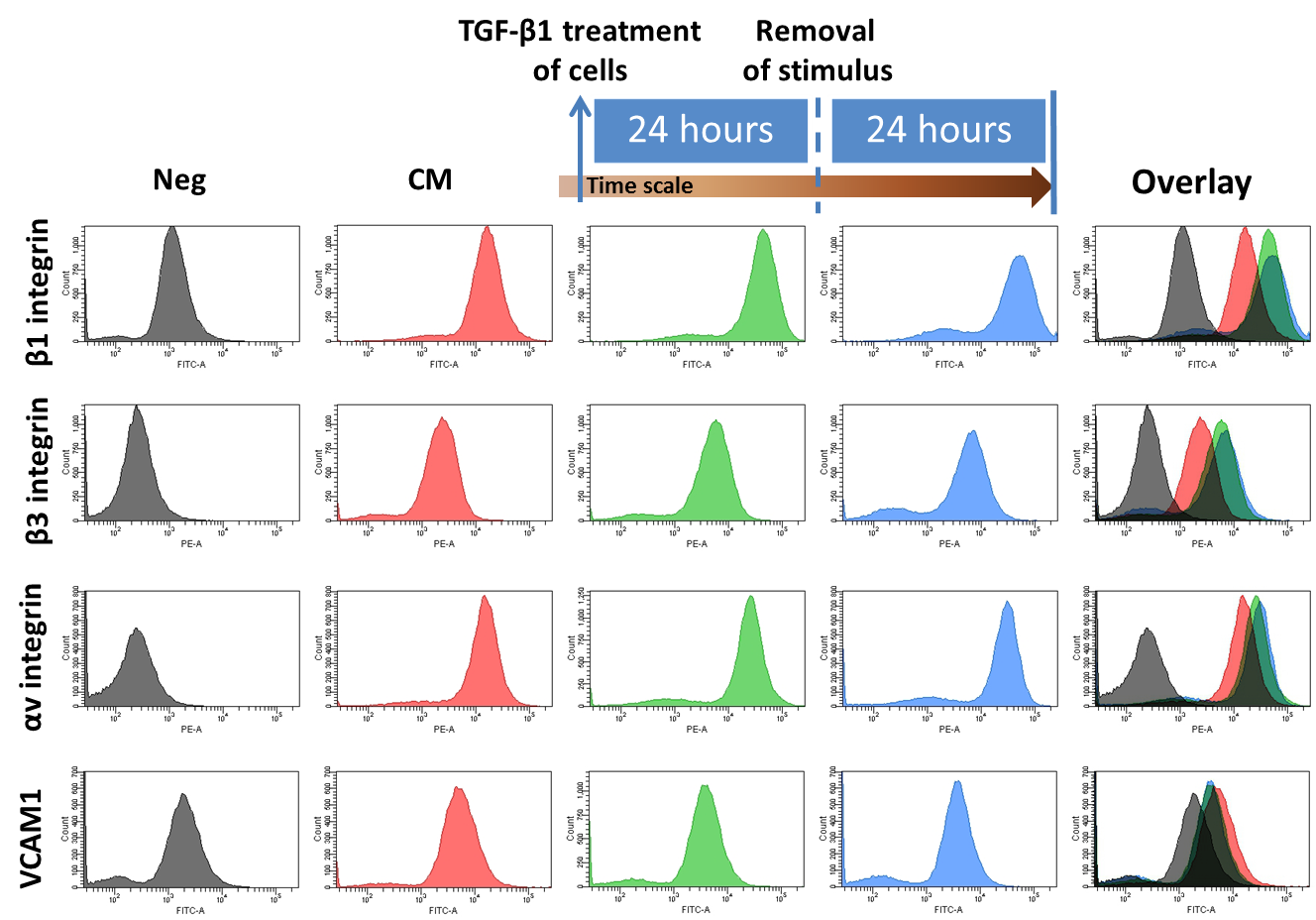


Supplementary Figure 5. Comparison of cell surface integrin expression between TGF-β1 treated MSCs at 24 hours and 48hours (additional 24 hours after removal of TGF-β1).

## Supplementary Tables

| Probe ID | Gene title | Gene symbol | TGF-Control |
| --- | --- | --- | --- |
| GROWTH FACTORS | | | |
| 1450759_at | bone morphogenetic protein 6 | Bmp6 | -4.14 |
| 1419139_at | growth differentiation factor 5 | Gdf5 | -3.01 |
| 1425379_at | hepatocyte growth factor | Hgf | -2.69 |
| 1438953_at | c-fos induced growth factor | Figf | -2.28 |
| 1422243_at | fibroblast growth factor 7 | Fgf7 | -2.12 |
| 1422912_at | bone morphogenetic protein 4 | Bmp4 | -1.90 |
| 1426319_at | platelet-derived growth factor, D polypeptide | Pdgfd | -1.65 |
| 1420847_a_at | fibroblast growth factor receptor 2 | Fgfr2 | -1.51 |
| 1418471_at | placental growth factor | Pgf | -1.48 |
| 1421441_at | angiopoietin 1 | Angpt1 | -1.47 |
| 1449873_at | bone morphogenetic protein 8a | Bmp8a | -1.42 |
| 1421916_at | platelet derived growth factor receptor, alpha polypeptide | Pdgfra | -1.29 |
| 1420795_at | fibroblast growth factor 9 | Fgf9 | -1.18 |
| 1423136_at | fibroblast growth factor 1 | Fgf1 | -1.06 |
| 1449545_at | fibroblast growth factor 18 | Fgf18 | -1.02 |
| 1423250_a_at | transforming growth factor, beta 2 | Tgfb2 | -0.64 |
| 1417455_at | transforming growth factor, beta 3 | Tgfb3 | 0.73 |
| 1452521_a_at | plasminogen activator, urokinase receptor | Plaur | 1.00 |
| 1451882_a_at | fibroblast growth factor 8 | Fgf8 | 1.01 |
| 1419417_at | vascular endothelial growth factor C | Vegfc | 1.05 |
| 1422916_at | fibroblast growth factor 21 | Fgf21 | 1.05 |
| 1420653_at | transforming growth factor, beta 1 | Tgfb1 | 1.08 |
| 1420085_at | fibroblast growth factor 4 | Fgf4 | 1.10 |
| 1450205_at | fibroblast growth factor 22 | Fgf22 | 1.12 |
| 1424932_at | epidermal growth factor receptor | Egfr | 1.26 |
| 1416623_at | thrombospondin 3 | Thbs3 | 1.27 |
| 1418596_at | fibroblast growth factor receptor 4 | Fgfr4 | 1.31 |
| 1421282_at | bone morphogenetic protein 5 | Bmp5 | 1.33 |
| 1420909_at | vascular endothelial growth factor A | Vegfa | 1.43 |
| 1418910_at | bone morphogenetic protein 7 | Bmp7 | 1.43 |
| 1422923_at | fibroblast growth factor 3 | Fgf3 | 1.44 |
| 1422019_at | transforming growth factor, beta receptor II | Tgfbr2 | 1.45 |
| 1415806_at | plasminogen activator, tissue | Plat | 1.57 |
| 1449826_a_at | fibroblast growth factor 2 | Fgf2 | 1.62 |
| 1421677_at | fibroblast growth factor 20 | Fgf20 | 1.85 |
| 1419519_at | insulin-like growth factor 1 | Igf1 | 2.38 |
| 1418711_at | platelet derived growth factor, alpha | Pdgfa | 2.52 |
| 1423635_at | bone morphogenetic protein 2 | Bmp2 | 3.86 |
| 1418350_at | heparin-binding EGF-like growth factor | Hbegf | 3.89 |
| probe id | **gene title** | **gene symbol** | **TGF-Control** |
| CYTOKINES AND CHEMOKINES | | | |
| 1419728_at | chemokine (C-X-C motif) ligand 5 | Cxcl5 | -8.60 |
| 1457644_s_at | chemokine (C-X-C motif) ligand 1 | Cxcl1 | -4.24 |
| 1415854_at | kit ligand | Kitl | -3.14 |
| 1417574_at | chemokine (C-X-C motif) ligand 12 | Cxcl12 | -2.96 |
| 1418126_at | chemokine (C-C motif) ligand 5 | Ccl5 | -2.48 |
| 1421404_at | chemokine (C-X-C motif) ligand 15 | Cxcl15 | -2.39 |
| 1422080_at | interleukin 7 | Il7 | -2.20 |
| 1415803_at | chemokine (C-X3-C motif) ligand 1 | Cx3cl1 | -2.18 |
| 1449399_a_at | interleukin 1 beta | Il1b | -2.14 |
| 1419530_at | interleukin 12b | Il12b | -1.95 |
| 1449990_at | interleukin 2 | Il2 | -1.79 |
| 1449990_at | interleukin 2 | Il2 | -1.79 |
| 1419529_at | interleukin 23, alpha subunit p19 | Il23a | -1.78 |
| 1417266_at | chemokine (C-C motif) ligand 6 | Ccl6 | -1.60 |
| 1421228_at | chemokine (C-C motif) ligand 7 | Ccl7 | -1.58 |
| 1421228_at | chemokine (C-C motif) ligand 7 | Ccl7 | -1.58 |
| 1423028_at | interferon alpha 2 | Ifna2 | -1.55 |
| 1431693_a_at | interleukin 17B | Il17b | -1.52 |
| 1421186_at | chemokine (C-C motif) receptor 2 | Ccr2 | -1.44 |
| 1421919_a_at | chemokine (C-C motif) receptor 9 | Ccr9 | -1.43 |
| 1417789_at | chemokine (C-C motif) ligand 11 | Ccl11 | -1.41 |
| 1422408_at | interferon alpha 4 | Ifna4 | -1.36 |
| 1438148_at | chemokine (C-X-C motif) ligand 3 | Cxcl3 | -1.30 |
| 1419697_at | chemokine (C-X-C motif) ligand 11 | Cxcl11 | -1.23 |
| 1421620_at | interleukin 5 receptor, alpha | Il5ra | -1.16 |
| 1425947_at | interferon gamma | Ifng | -1.08 |
| 1417936_at | chemokine (C-C motif) ligand 9 | Ccl9 | -1.06 |
| 1418718_at | chemokine (C-X-C motif) ligand 16 | Cxcl16 | 1.01 |
| 1421608_at | interleukin 20 | Il20 | 1.04 |
| 1422053_at | inhibin beta-A | Inhba | 1.06 |
| 1449864_at | interleukin 4 | Il4 | 1.06 |
| 1418803_a_at | Fas ligand (TNF superfamily, member 6) | Fasl | 1.13 |
| 1449277_at | chemokine (C-C motif) ligand 19 | Ccl19 | 1.15 |
| 1421688_a_at | chemokine (C-C motif) ligand 1 | Ccl1 | 1.22 |
| 1421034_a_at | interleukin 4 receptor, alpha | Il4ra | 1.27 |
| 1422259_a_at | chemokine (C-C motif) receptor 5 | Ccr5 | 1.84 |
| 1421473_at | interleukin 1 alpha | Il1a | 1.85 |
| 1422305_at | interferon beta 1, fibroblast | Ifnb1 | 1.91 |
| 1421207_at | leukemia inhibitory factor | Lif | 2.32 |
| Probe ID | **Gene title** | **Gene symbol** | **TGF-Control** |
| 1449982_at | interleukin 11 | Il11 | 3.22 |
| 1448710_at | chemokine (C-X-C motif) receptor 4 | Cxcr4 | 3.46 |
| 1422812_at | chemokine (C-X-C motif) receptor 6 | Cxcr6 | 5.92 |
| EXTRACELLULAR MATRIX | | | |
| 1429072_at | collagen, type VI, alpha 4 | Col6a4 | -4.37 |
| 1450798_at | tenascin XB | Tnxb | -2.94 |
| 1420484_a_at | vitronectin | Vtn | -2.67 |
| 1435386_at | Von Willebrand factor homolog | Vwf | -1.29 |
| 1450567_a_at | collagen, type II, alpha 1 | Col2a1 | -1.09 |
| 1425772_at | collagen, type IV, alpha 4 | Col4a4 | -0.94 |
| 1448291_at | matrix metallopeptidase 9 | Mmp9 | -0.89 |
| 1425475_at | collagen, type IV, alpha 5 | Col4a5 | -0.88 |
| 1460227_at | tissue inhibitor of metalloproteinase 1 | Timp1 | 0.66 |
| 1441506_at | decorin | Dcn | 0.81 |
| 1423669_at | collagen, type I, alpha 1 | Col1a1 | 0.82 |
| 1439364_a_at | matrix metallopeptidase 2 | Mmp2 | 0.92 |
| 1446326_at | collagen, type I, alpha 2 | Col1a2 | 0.95 |
| 1450625_at | collagen, type V, alpha 2 | Col5a2 | 0.99 |
| 1427009_at | laminin, alpha 5 | Lama5 | 1.02 |
| 1427512_a_at | laminin, alpha 3 | Lama3 | 1.05 |
| 1423578_at | collagen, type XI, alpha 2 | Col11a2 | 1.12 |
| 1421006_at | collagen, type IV, alpha 6 | Col4a6 | 1.17 |
| 1424807_at | laminin, alpha 4 | Lama4 | 1.20 |
| 1419703_at | collagen, type V, alpha 3 | Col5a3 | 1.28 |
| 1427884_at | collagen, type III, alpha 1 | Col3a1 | 1.31 |
| 1426642_at | fibronectin 1 | Fn1 | 1.33 |
| 1418599_at | collagen, type XI, alpha 1 | Col11a1 | 1.42 |
| 1421694_a_at | versican | Vcan | 1.43 |
| 1450224_at | collagen, type IV, alpha 3 | Col4a3 | 1.55 |
| 1425594_at | laminin gamma 3 | Lamc3 | 2.00 |
| 1416741_at | collagen, type V, alpha 1 | Col5a1 | 2.31 |
| 1416342_at | tenascin C | Tnc | 5.43 |
| 1423606_at | periostin, osteoblast specific factor | Postn | 6.72 |
| ADHESION MOLECULES (INTEGRINS) | | | |
| 1425367_at | integrin alpha L | Itgal | -2.47 |
| 1420860_at | integrin alpha 9 | Itga9 | -1.92 |
| 1418741_at | integrin beta 7 | Itgb7 | -1.86 |
| 1439713_at | integrin alpha 1 | Itga1 | -1.62 |
| 1421997_s_at | integrin alpha 3 | Itga3 | -1.14 |
| 1427489_at | integrin alpha 8 | Itga8 | -1.08 |
| 1450501_at | integrin alpha 2 | Itga2 | 0.54 |
| probe id | **gene title** | **gene symbol** | **TGF-Control** |
| 1422983_at | integrin beta 6 | Itgb6 | 0.60 |
| 1417534_at | integrin beta 5 | Itgb5 | 0.86 |
| 1421194_at | integrin alpha 4 | Itga4 | 0.91 |
| 1433053_at | integrin beta 2-like | Itgb2l | 1.05 |
| 1418393_a_at | integrin alpha 7 | Itga7 | 1.09 |
| 1421198_at | integrin alpha V | Itgav | 1.12 |
| 1419128_at | integrin alpha X | Itgax | 1.28 |
| 1426918_at | integrin beta 1 (fibronectin receptor beta) | Itgb1 | 1.36 |
| 1423267_s_at | integrin alpha 5 (fibronectin receptor alpha) | Itga5 | 1.65 |
| 1421511_at | integrin beta 3 | Itgb3 | 1.83 |
| ADHESION MOLECULES (OTHERS) | | | |
| 1449563_at | contactin 1 | Cntn1 | -4.09 |
| 1415989_at | vascular cell adhesion molecule 1 | Vcam1 | -3.89 |
| 1440220_at | occludin | Ocln | -2.38 |
| 1430583_at | neuron-glia-CAM-related cell adhesion molecule | Nrcam | -2.22 |
| 1426673_at | cadherin 3 | Cdh3 | -1.75 |
| 1421592_at | neural cell adhesion molecule 2 | Ncam2 | -1.62 |
| 1421958_at | L1 cell adhesion molecule | L1cam | -1.10 |
| 1421966_at | neural cell adhesion molecule 1 | Ncam1 | -1.05 |
| 1448261_at | cadherin 1 | Cdh1 | -1.03 |
| 1418815_at | cadherin 2 | Cdh2 | 0.90 |
| 1421712_at | selectin, endothelial cell | Sele | 0.95 |
| 1426300_at | activated leukocyte cell adhesion molecule | Alcam | 1.03 |
| 1420853_at | syndecan 3 | Sdc3 | 1.09 |
| 1418921_at | cell adhesion molecule 3 | Cadm3 | 1.16 |
| 1420558_at | selectin, platelet | Selp | 1.43 |
| 1449422_at | cadherin 4 | Cdh4 | 1.96 |
| 1435165_at | contactin 2 | Cntn2 | 4.96 |
| CYTOSKELETON | | | |
| 1436042_at | talin 1 | Tln1 | -1.40 |
| 1417558_at | Fyn proto-oncogene | Fyn | 0.61 |
| 1416156_at | vinculin | Vcl | 0.66 |
| 1420805_at | myosin, light chain 10, regulatory | Myl10 | 0.72 |
| 1425978_at | myocardin | Myocd | 0.76 |
| 1420979_at | p21 protein (Cdc42/Rac)-activated kinase 1 | Pak1 | 0.85 |
| 1434653_at | PTK2 protein tyrosine kinase 2 beta | Ptk2b | 0.90 |
| 1415927_at | actin, alpha, cardiac muscle 1 | Actc1 | 0.97 |
| 1427385_s_at | actinin, alpha 1 | Actn1 | 1.11 |
| 1440981_at | p21 protein (Cdc42/Rac)-activated kinase 7 | Pak7 | 1.35 |
| 1429111_at | talin 2 | Tln2 | 1.40 |
| 1452670_at | myosin, light polypeptide 9, regulatory | Myl9 | 1.65 |
| 1441057_at | Myosin, heavy polypeptide 10, non-muscle | Myh10 | 1.79 |
| probe id | **gene title** | **gene symbol** | **TGF-Control** |
| TRANSCRIPTION FACTORS AND OTHER SIGNALING MOLECULES | | | |
| 1422602_a_at | wingless-related MMTV integration site 5B | Wnt5b | -3.46 |
| 1423259_at | inhibitor of DNA binding 4 | Id4 | -3.40 |
| 1425895_a_at | inhibitor of DNA binding 1 | Id1 | -2.55 |
| 1416630_at | inhibitor of DNA binding 3 | Id3 | -2.16 |
| 1421964_at | Notch gene homolog 3 (Drosophila) | Notch3 | -1.63 |
| 1433471_at | transcription factor 7, T-cell specific | Tcf7 | -1.55 |
| 1422938_at | B-cell leukemia/lymphoma 2 | Bcl2 | -1.51 |
| 1419301_at | frizzled homolog 4 (Drosophila) | Fzd4 | -1.44 |
| 1422537_a_at | inhibitor of DNA binding 2 | Id2 | -1.43 |
| 1421465_at | wingless related MMTV integration site 2b | Wnt2b | -1.41 |
| 1450782_at | wingless-related MMTV integration site 4 | Wnt4 | -1.37 |
| 1422937_at | frizzled homolog 5 (Drosophila) | Fzd5 | -1.32 |
| 1422771_at | MAD homolog 6 (Drosophila) | Smad6 | -1.31 |
| 1425447_at | dickkopf homolog 4 (Xenopus laevis) | Dkk4 | -1.27 |
| 1434439_at | glycogen synthase kinase 3 beta | Gsk3b | -1.17 |
| 1423367_at | wingless-related MMTV integration site 7A | Wnt7a | -1.08 |
| 1429045_at | SMAD specific E3 ubiquitin protein ligase 2 | Smurf2 | -0.67 |
| 1421047_at | MAD homolog 5 (Drosophila) | Smad5 | 0.58 |
| 1450471_at | MAD homolog 3 (Drosophila) | Smad3 | 0.58 |
| 1443005_at | zinc finger E-box binding homeobox 1 | Zeb1 | 0.63 |
| 1423389_at | MAD homolog 7 (Drosophila) | Smad7 | 0.72 |
| 1418633_at | Notch gene homolog 1 (Drosophila) | Notch1 | 0.94 |
| 1421299_a_at | lymphoid enhancer binding factor 1 | Lef1 | 1.03 |
| 1448742_at | snail homolog 1 (Drosophila) | Snai1 | 1.16 |
| 1436901_at | Notch gene homolog 4 (Drosophila) | Notch4 | 1.17 |
| 1450043_at | frizzled homolog 7 (Drosophila) | Fzd7 | 1.18 |
| 1426091_a_at | wingless related MMTV integration site 10b | Wnt10b | 1.38 |
| 1426430_at | jagged 2 | Jag2 | 1.46 |
| 1418532_at | frizzled homolog 2 (Drosophila) | Fzd2 | 1.65 |
| 1450772_at | wingless-related MMTV integration site 11 | Wnt11 | 1.75 |
| 1440182_at | frizzled homolog 10 (Drosophila) | Fzd10 | 1.91 |
| 1423379_at | nuclear factor of activated T-cells, cytoplasmic, calcineurin-dependent 4 | Nfatc4 | 1.92 |
| 1422300_at | noggin | Nog | 2.18 |
| 1417621_at | nuclear factor of activated T-cells, cytoplasmic, calcineurin-dependent 1 | Nfatc1 | 2.33 |
| 1417301_at | frizzled homolog 6 (Drosophila) | Fzd6 | 2.48 |
| 1420512_at | dickkopf homolog 2 (Xenopus laevis) | Dkk2 | 2.48 |
| 1421439_at | wingless related MMTV integration site 8b | Wnt8b | 2.51 |
| 1425901_at | nuclear factor of activated T-cells, cytoplasmic, calcineurin-dependent 2 | Nfatc2 | 3.76 |
| 1421341_at | axin2 | Axin2 | 4.00 |
| 1420891_at | wingless-related MMTV integration site 7B | Wnt7b | 4.60 |
| 1425889_at | wingless-type MMTV integration site 9A | Wnt9a | 4.63 |

**Supplementary Table 1.** Regulation of critical genes related to MSC function and wound healing reported as log2 ratio.

# References

1. McGrail, D. J., Ghosh, D., Quach, N. D. & Dawson, M. R. Differential Mechanical Response of Mesenchymal Stem Cells and Fibroblasts to Tumor-Secreted Soluble Factors. *PLoS One* **7,** e33248 (2012).

2. Ghosh, D., Lili, L., McGrail, D. J., Matyunina, L. V, McDonald, J. F. & Dawson, M. R. Integral role of platelet-derived growth factor in mediating transforming growth factor-β1-dependent mesenchymal stem cell stiffening. *Stem Cells Dev.* **23,** 245–261 (2014).
